# Lisinopril-Induced Angioedema Triggered by Tattoo Cellulitis and Acute Kidney Injury: A Case Report

**DOI:** 10.7759/cureus.101391

**Published:** 2026-01-12

**Authors:** Mechal Zedan, Stephen Howard, Preston Foryt, Tarilate Aroriode

**Affiliations:** 1 Internal Medicine, Lake Erie College of Osteopathic Medicine, Rochester, USA; 2 Physical Medicine and Rehabilitation, Rochester Regional Health, Rochester, USA; 3 Nursing, St. John Fisher University, Rochester, USA; 4 Internal Medicine, Rochester Regional Health, Rochester, USA

**Keywords:** ace inhibitor induced angioedema, acute kidney injury, bradykinin mediated angioedema, cellulitis, tattoo

## Abstract

Angiotensin-converting enzyme inhibitor-induced angioedema is a rare, potentially life-threatening disorder. This mostly manifests with swelling of the lips, tongue, and face with no known reports of angiotensin-converting enzyme inhibitor-induced angioedema from soft-tissue infections. We present herein a case of a 47-year-old male with a history of chronic kidney disease stage III who developed acute upper lip angioedema and severe acute kidney injury while on lisinopril/hydrochlorothiazide, following tattoo-related cellulitis. We propose that inflammatory activation from cellulitis amplified kallikrein-kinin system activity and bradykinin generation, precipitating angioedema in the setting of angiotensin-converting enzyme inhibition and transient renal dysfunction. This case highlights a rare but important intersection between infection-driven inflammation and angiotensin-converting enzyme inhibitor-induced angioedema.

## Introduction

Angioedema is a potentially life-threatening adverse effect of angiotensin-converting enzyme (ACE) inhibitors, resulting from impaired degradation of bradykinin and increased vascular permeability [1]. It can develop unpredictably, even after years of therapy. Systemic inflammation and certain medications such as non-steroidal anti-inflammatory drugs, beta lactams, and dipeptidyl peptidase-4 inhibitors have been linked to increased risk of ACE inhibitor-related angioedema, but there are no reports of soft-tissue infections triggering this adverse effect [2,3].

ACE inhibitor-induced angioedema occurs in approximately 0.1-0.7% of users and involves a bradykinin-mediated process which is distinct from allergic (histaminergic) angioedema [4]. ACE inhibition reduces degradation of bradykinin and substance P, leading to accumulation of these vasoactive peptides [5]. Bradykinin binds to B2 receptors on endothelial cells, activating phospholipase A2 and phospholipase C pathways which leads to production of nitric oxide, prostacyclin, and prostaglandins, resulting in local vasodilation and increased vascular permeability [6]. Clinically, swelling most often affects the lips, tongue, and face, usually without pruritus or urticaria, and typically does not respond to antihistamines, corticosteroids, or epinephrine [7-9].

## Case presentation

**Figure 1:**
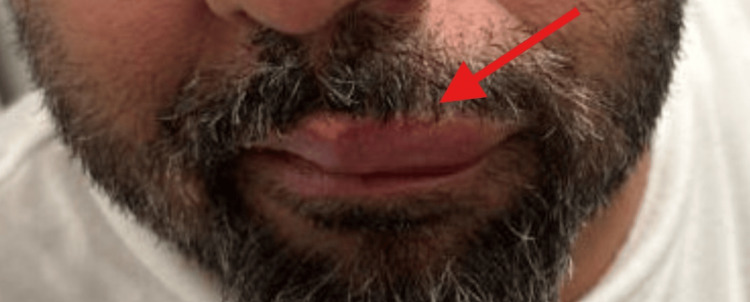
Left-sided upper lip swelling The red arrow on the patients left upper lip indicates the swelling experienced on presentation to the emergency department.

**Figure 2:**
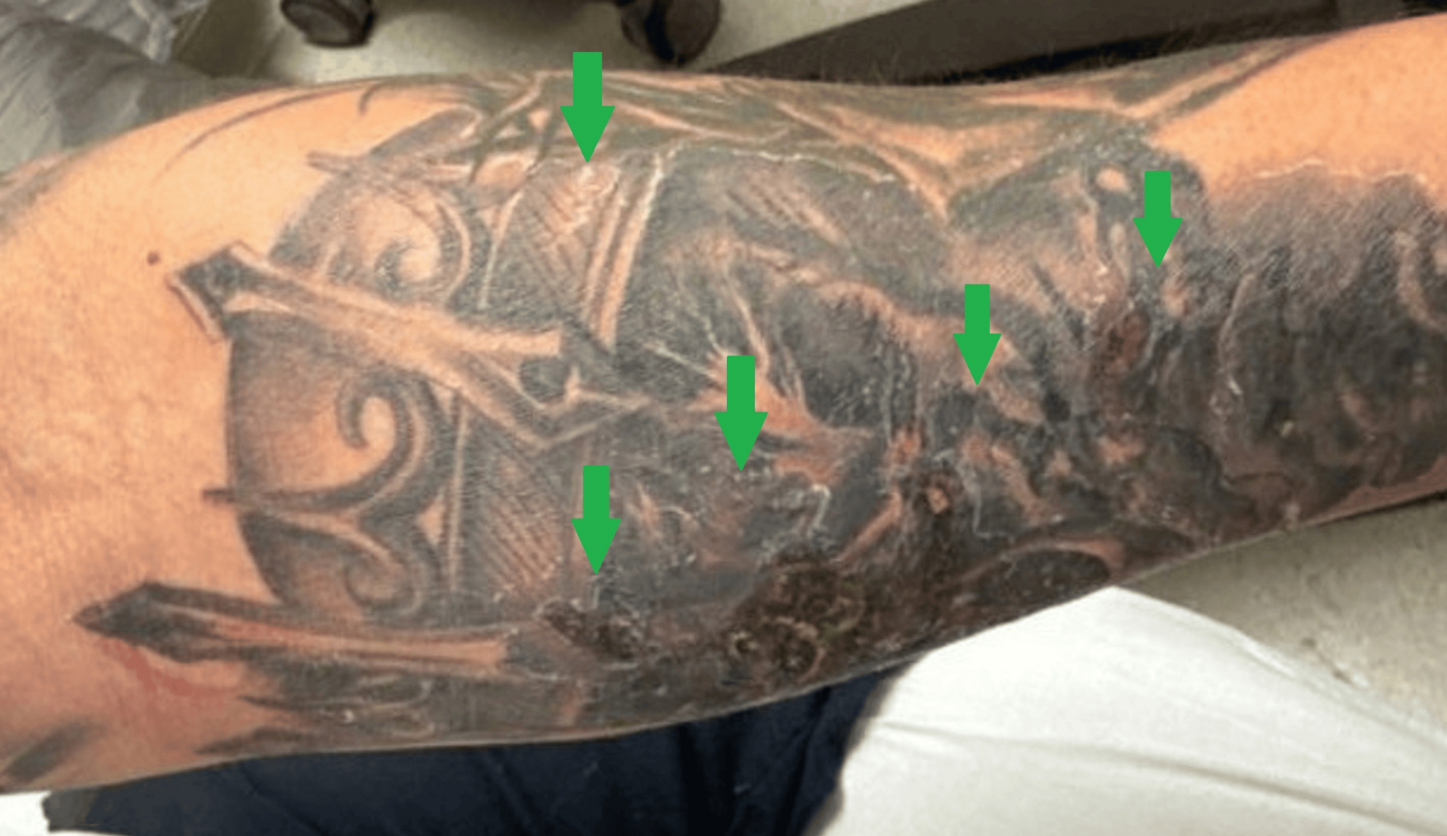
Left arm erythema and scaling at tattoo site The green arrows indicate the patient's scaling skin after tattoo placement with surrounding erythema.

A 47-year-old male with chronic kidney disease (CKD) stage III and hypertension on lisinopril-hydrochlorothiazide presented with sudden-onset swelling of the left upper lip. He denied dyspnea, dysphonia, or previous similar episodes. He had taken lisinopril for 10 years and switched to combination therapy (lisinopril + hydrochlorothiazide) six months earlier. One week before presentation, he received a tattoo on his left forearm, which became erythematous, pruritic, and later pustular, with low-grade fever two days post-procedure. On arrival, his vitals were stable. Physical examination revealed asymmetric upper and lower lip edema (Figure 1) and erythematous scaling of the left forearm tattoo site (Figure 2). Labs were significant for mild leukocytosis (WBC 11.2 ×10³cells per microliter), blood urea nitrogen of 71 milligrams per deciliter, estimated glomerular filtration rate (eGFR) of 5 milliliter per minute (normal > 60 milliliter per minute), and creatinine of 11.2 milligrams per decilitre (his baseline is 1.6 milligrams per deciliter). Non-contrast CT scan of the abdomen and pelvis revealed mild right hydronephrosis without obstruction. 

He received diphenhydramine in the ED with partial improvement. ENT evaluation confirmed no airway compromise. Lisinopril was discontinued, and doxycycline and amoxicillin-clavulanate were started for cellulitis. His lip swelling resolved within 48 hours. Creatinine gradually improved to 1.9 milligrams per deciliter over 10 days. Kidney biopsy revealed acute tubular injury with mild focal interstitial inflammation, consistent with acute kidney injury (AKI) on CKD.

## Discussion

This case underscores a rare systemic interaction between localized inflammatory infection (tattoo cellulitis) and ACE inhibitor-induced angioedema. The temporal relationship between infection, renal dysfunction, and orofacial swelling suggests a convergence of inflammatory, endothelial, and metabolic pathways that amplify bradykinin-mediated vascular permeability. The contact system, also known as the kallikrein-kinin system, is a plasma protease cascade that links coagulation, inflammation, and innate immunity. Its main function is to generate bradykinin, a potent inflammatory mediator that increases vascular permeability, vasodilation, pain, and edema [10]. The contact system is activated when factor XII (also known as Hageman factor) comes into contact with negatively charged or foreign surfaces, such as bacterial components, or extracellular matrix fragments. Activated FXII (FXIIa) then converts prekallikrein to kallikrein, which in turn cleaves high-molecular-weight kininogen to release bradykinin, the potent vasodilator implicated in angioedema. Normally, C1 esterase inhibitor (C1-INH) keeps the contact system in check by inhibiting FXIIa and kallikrein. Its deficiency is the main cause of hereditary angioedema [11] and it is here where the role of the complement system in the genesis of ACE inhibitor-associated angioedema comes into play.

In the setting of a soft tissue infection, the disruption of endothelial integrity and activation of innate immunity drives complement activation, which leads to increased binding of C1-INH to activated C1 complexes and other serine proteases, thereby consuming the effective functional reserve of C1-INH [12]. A small study in 1997 examining the levels of carboxypeptidase N and complement components as potential predictors of ACE inhibitor-associated angioedema found that ACE inhibitor-associated angioedema patients had significantly lower levels of carboxypeptidase N (also known as kininase I) and C1-INH compared with controls [13]. This consumption of C1-INH may lead to reduced regulation of contact system activation resulting in increased kallikrein-mediated release of bradykinin [12]. At the same time, ACE inhibitor therapy impairs bradykinin degradation (blocking kininase II), resulting in bradykinin accumulation even when production is only modestly elevated [5]. Thus, in the infected patient on ACE inhibitors, the triad of complement-driven C1-INH consumption, contact system activation with bradykinin generation, and reduced bradykinin breakdown converge to trigger a bradykinin-mediated angioedema event. It is well established that various forms of infections, including cellulitis, can precipitate AKI, and observational data show a clear association between acute infections and transient AKI episodes in patients taking antihypertensives, including ACE inhibitors, while infection-associated AKI is common in ACE inhibitor users [14]. However, its mechanistic relevance to angioedema here is subtler. Once AKI occurs, injured renal cells amplify complement activation and reduce clearance of complement fragments, creating a vicious cycle of complement activation [15,16]. Therefore, we propose that this excessive complement activity could potentially rapidly increase C1-INH consumption, and in patients taking ACE inhibitors, who already have impaired bradykinin breakdown, this could possibly allow uncontrolled contact system activity. The result is increased bradykinin production and accumulation and exaggerated vascular permeability, worsening the severity of ACE inhibitor-associated angioedema.

## Conclusions

This case highlights how localized inflammatory infection can act as a systemic stressor, triggering ACE inhibitor-induced angioedema through activation of the contact system, the complement system, and enhanced endothelial reactivity. In this setting, a superimposed transient AKI could theoretically lead to amplified complement system activity with the subsequent consumption of C1-INH. This interaction between contact system activation, complement system activation enhanced by AKI, transient C1-esterase inhibitory deficiency, and impaired bradykinin degradation due to ACE inhibitor therapy emphasizes the main underlying mechanisms leading to angioedema in patients taking ACE inhibitors with concurrent AKI and soft tissue infections. Clinicians should maintain vigilance for angioedema in ACE inhibitor users presenting with new infections, inflammatory conditions, or trauma, even when the affected region is anatomically distant from the site of swelling.
